# Bloody Stools in the Emergency Room: Cefdinir-Induced Red Stools in a 7-Month-Old Male

**DOI:** 10.7759/cureus.68887

**Published:** 2024-09-07

**Authors:** Ali Shammout, Philip Pazderka

**Affiliations:** 1 Emergency Medicine, Western Michigan University Homer Stryker M.D. School of Medicine, Kalamazoo, USA

**Keywords:** pediatric infection, emergency gastroenterology and endoscopy, emergency medicine, cefdinir, bloody diarrhea

## Abstract

This case report presents a 7-month-old male who was admitted to the emergency room with red-colored stools, initially raising concerns for serious gastrointestinal issues. The child, who had a history of milk protein allergy and eczema, had recently been prescribed cefdinir for an ear infection and was also consuming a hydrolyzed formula containing iron. Despite initial findings of elevated white blood cell count, mild anemia, and hyperkalemia, a stool heme-occult test was negative. The negative heme-occult lead to the consideration of cefdinir-induced stool discoloration as a possible diagnosis, a benign side effect that occurs in the presence of iron supplementation. Following the discontinuation of cefdinir, the patient’s symptoms resolved completely on follow up with his pediatrician. A rare occurrence, cefdinir-induced red stool discoloration must be considered in cases of benign appearing infants with “bloody” stools.

## Introduction

The presence of bloody stools in young pediatric patients can pose a diagnostic challenge requiring consideration of common and rare causes. While the initial workup for red-colored stools often considers common causes like gastroenteritis, intussusception, necrotizing enterocolitis, or anatomical abnormalities, rarer etiologies may require a more comprehensive history and a range of diagnostic tools from laboratory tests to upper or lower endoscopies [1]. Cefdinir, a third generation cephalosporin, is commonly used in pediatric patients for the treatment of a broad range of infectious pathologies [2]. While generally considered safe and strongly effective, cefdinir can present with a modicum of different side effects. In this case, we present a 7-month-old patient who arrived at the emergency room with bloody stools, ultimately diagnosed as cefdinir-induced red stool discoloration.

## Case presentation

A 7-month-old male with a history of milk protein allergy and eczema presented to the emergency department with multiple episodes of red-colored stools. The patient had previously experienced bloody stools due to his milk protein allergy and was switched to a hydrolyzed formula months earlier. He maintained a good appetite, consuming five to six bottles daily, with no signs of dehydration on physical examination. There were no abnormal vital signs, and the abdominal examination revealed a non-tender, non-distended abdomen. Laboratory results showed an elevated white blood cell count of 17.9, hemoglobin of 11.2, potassium of 5.7, and calcium of 11.1, with a notably negative heme-occult test for stool (Table 1). 

**Table 1:** Patient’s laboratory values upon presentation to the Emergency Department with corresponding reference ranges. BUN: Blood Urea Nitrogen; Calcium: Calcium; HGB: Haemoglobin; HCT: Haematocrit; MCV: Mean Corpuscular Volume; MCH: Mean Corpuscular Haemoglobin; MCHC: Mean Corpuscular Haemoglobin Concentration; RDW: Red Cell Distribution Width; MPV: Mean Platelet Volume.

Parameters	Patient Values	Reference Range
Glucose	91	70-99 mg/dL
Sodium	139	135-145 mmol/L
Potassium	5.7	3.5-5.3 mmol/L
Chloride	104	98-108 mmol/L
CO2	20	23-32 mmol/L
Anion Gap	15	7-16 mmol/L
Creatinine	0.13	0.2-0.7 mg/dL
BUN	12	4-19 mg/dL
Calcium	11.1	8.6-10.3 mg/dL
WBC	17.9	6-17.5 10*9/L
RBC	4.22	3.7-5.3 10*12/L
HGB	11.2	10.5-14 g/dL
HCT	34.30%	33.0-42.0%
MCV	81.3	70-88 fL
MCH	26.5	25-35 pg/cell
MCHC	32.7	32-36 g/dL
RDW	13.40%	<15%
Platelet Count	356	140-440 10*9/L
MPV	9.2	9-12.2 fL

Further history revealed that the patient had been diagnosed with a right ear infection the previous day and was prescribed cefdinir. After consultation with the pediatric hospitalist, pediatric surgery service, and emergency room pharmacy, it was decided to discontinue cefdinir, administer a single intramuscular injection of ceftriaxone, and discharge the patient with strict return precautions. A literature review during the clinical evaluation mentioned potential red discoloration of stools due to cefdinir interacting with iron supplementation. The patient’s hydrolyzed formula contained 1.8 mg of iron per serving. Following the discontinuation of cefdinir, the patient reported complete resolution of the red stool discoloration at his follow-up visit the following day.

## Discussion

Cefdinir is a broad-spectrum antibiotic commonly used to treat various bacterial infections in pediatric patients, such as pharyngitis and otitis media, and is generally well-tolerated [3]. However, case reports have noted instances of "bloody diarrhea" associated with cefdinir use, with fewer than ten cases documented in the current literature [3]. These cases often involve concurrent oral iron supplementation, as seen in our patient [4]. Patients are usually prescribed cefdinir for the management of otitis media, receive a range of different dosages of the cefdinir, and are typically less than a year old [5]. The presentation is typically without other gastrointestinal symptoms, such as vomiting, abdominal pain, or a history of diarrhea [6].

This lack of additional gastrointestinal or systemic symptoms can help differentiate cefdinir-induced red stool discoloration from more significant pathological causes, like bacterial gastroenteritis [7]. Standard workup for suspected bloody diarrhea can include stool heme-occult tests, microbial cultures, toxin assays, and imaging studies such as abdominal radiographs [8]. As in our patient, cases throughout the literature suggest a negative fecal occult blood test across each of the documented patients [6]. The negative fecal occult blood test may itself serve as a possible sign for suspecting cefdinir-induced “bloody” stools, yet this sign must be studied further as, in the current literature, there is an ongoing discussion on the viability of usage of fecal occult blood test in the acute setting [9]. To avoid unnecessary diagnostics and treatments, recognizing Cefdinir as a potential cause of red stool discoloration is crucial, especially in patients with recent cefdinir use and ongoing iron supplementation [8]. Furthermore, initial education of parents who are receiving cefdinir prescribed for their child by the physicians managing these children may alleviate panic-filled situations and limit visitations to the emergency room as many patients are not aware of this side effect of the commonly used medication [10].

Management of the patients after cefdinir-induced “bloody” stools can range based on the provider and the situation. Some case reports describe the ongoing continuation of the antibiotic with proper education given to the parents and no report of any subsequent sequelae of continuing the medication [11]. Other providers, such as the ones responsible for the patient we presented in our case, preferred a switch to a different antibiotic, such as intramuscular ceftriaxone, to halt the worrying stools and provide definitive care to the patient. Regardless of the method decided upon, the presentation is benign; the focus should revolve around treating the patient and providing reassurance to the parents.

## Conclusions

This case underscores the importance of thorough history-taking and considering less common causes of red-colored stools in pediatric patients. The diagnosis of cefdinir-induced red stool discoloration, particularly with concurrent iron supplementation, highlights the need for awareness of this benign etiology. Recognizing this adverse effect can prevent unnecessary diagnostic procedures and interventions, allowing for appropriate clinical management and patient education. Additionally, a multidisciplinary approach involving pediatricians, pharmacists, and specialists is essential for comprehensive and accurate patient care. Prompt recognition and management of cefdinir-related stool discoloration can alleviate concerns and provide reassurance to healthcare providers and families alike.
